# Author Correction: Cytokine release syndrome-like serum responses after COVID-19 vaccination are frequent and clinically inapparent under cancer immunotherapy

**DOI:** 10.1038/s43018-022-00420-y

**Published:** 2022-07-15

**Authors:** Thomas Walle, Sunanjay Bajaj, Joscha A. Kraske, Thomas Rösner, Christiane S. Cussigh, Katharina A. Kälber, Lisa Jasmin Müller, Sophia Boyoung Strobel, Jana Burghaus, Stefan M. Kallenberger, Christoph K. Stein-Thöringer, Maximilian Jenzer, Antonia Schubert, Steffen Kahle, Anja Williams, Birgit Hoyler, Lin Zielske, Renate Skatula, Stefanie Sawall, Mathias F. Leber, Russell Z. Kunes, Johannes Krisam, Carlo Fremd, Andreas Schneeweiss, Jürgen Krauss, Leonidas Apostolidis, Anne Katrin Berger, Georg M. Haag, Stefanie Zschäbitz, Niels Halama, Christoph Springfeld, Romy Kirsten, Jessica C. Hassel, Dirk Jäger, NCT ANTICIPATE Investigators, Omar Abdelrahim, Elena Busch, Patrick Derigs, Katharina Dischinger, Fouad Mitri, Kerstin Schmidt, Irfan A. Bhatti, Barbara Grün, Nicolas Hohmann, Lena Woydack, Xin-Wen Zhang, Dyke Ferber, Andreas Mock, Tillmann Pompecki, Timo Schank, Carlo Fremd, Georg M. Haag, Niels Halama, Romy Kirsten, Jessica C. Hassel, Dirk Jäger, Guy Ungerechts

**Affiliations:** 1 Clinical Cooperation Unit Virotherapy, German Cancer Research Center (DKFZ), Heidelberg, Germany; 2 Department of Medical Oncology, National Center for Tumor Diseases, Heidelberg University Hospital, Heidelberg, Germany; 3 German Cancer Consortium (DKTK), Heidelberg, Germany; 4 Department of Dermatology, National Center for Tumor Diseases, Heidelberg University Hospital, Heidelberg, Germany; 5 Department of Hematology, University Hospital Heidelberg, Heidelberg, Germany; 6 Division Microbiome and Cancer, German Cancer Research Center (DKFZ), Heidelberg, Germany; 7 BioQuant & Department of Cell and Molecular Biology, Heidelberg University, Heidelberg, Germany; 8 Division Signaling and Functional Genomics, German Cancer Research Center (DKFZ), Heidelberg, Germany; 9 NCT Liquid Biobank, National Center for Tumor Diseases (NCT), Heidelberg, Germany; 10 Department of Statistics, Columbia University, New York, NY USA; 11 Institute of Medical Biometry, Heidelberg University, Heidelberg, Germany; 12 Division of Gynecological Oncology, National Center for Tumor Diseases (NCT), Heidelberg, Germany; 13 Clinical Cooperation Unit Applied Tumor-Immunity, German Cancer Research Center (DKFZ), Heidelberg, Germany; 14 Department of Translational Immunotherapy, German Cancer Research Center (DKFZ), Heidelberg, Germany; 15 Helmholtz Institute for Translational Oncology (HITRON), Mainz, Germany; 16 CanVirex, Heidelberg, Germany; 17 Ottawa Hospital Research Institute, Cancer Therapeutics Program, Ottawa, Ontario Canada; 18 Department of Gastroenterology, Heidelberg University Hospital, Heidelberg, Germany

**Keywords:** SARS virus, Viral infection, Cancer, Cancer immunotherapy

Correction to: *Nature Cancer* 10.1038/s43018-022-00398-7, published online 17 June 2022.

In the version of this article initially published, salient aspects of patient outcomes were not clearly articulated in the abstract, which has now been revised to include the text “Within 4 weeks after vaccination, serious adverse events occurred in eight patients (12.5% 95% CI 5.6–23%): six patients were hospitalized due to events common under cancer therapy including immune related adverse events and two patients died due to conditions present before vaccination”. The changes have been made in the HTML and PDF versions of the article.

